# Analysis of the potential effect of ponatinib on the QTc interval in patients with refractory hematological malignancies

**DOI:** 10.1007/s00280-013-2160-7

**Published:** 2013-04-23

**Authors:** Daryl Sonnichsen, David J. Dorer, Jorge Cortes, Moshe Talpaz, Michael W. Deininger, Neil P. Shah, Hagop M. Kantarjian, Dale Bixby, Michael J. Mauro, Ian W. Flinn, Jeffrey Litwin, Christopher D. Turner, Frank G. Haluska

**Affiliations:** 1 ARIAD Pharmaceuticals, Inc., 26 Landsdowne St., Cambridge, MA 02139 USA; 2 Sonnichsen Pharmaceutical Associates, Collegeville, PA USA; 3 Division of Cancer Medicine, Department of Leukemia, The University of Texas M.D. Anderson Cancer Center, Houston, TX USA; 4 Division of Hematology and Oncology, Department of Internal Medicine, University of Michigan Comprehensive Cancer Center, Ann Arbor, MI USA; 5 Center for Hematologic Malignancies, Knight Cancer Institute, Oregon Health & Science University, Portland, OR USA; 6 Department of Hematology/Oncology, University of California San Francisco, San Francisco, CA USA; 7 Division of Hematology and Oncology, University of Michigan Comprehensive Cancer Center, Ann Arbor, MI USA; 8 Data Safety Monitoring Committee, Center for Hematologic Malignancies, Knight Cancer Institute, Oregon Health and Science University, Portland, OR USA; 9 Hematologic Malignancies Research Program, Sarah Cannon Research Institute, Nashville, TN USA; 10 ERT Inc., Philadelphia, PA USA; 12 Present Address: Division of Hematology and Hematologic Malignancies, Department of Oncological Sciences, Huntsman Cancer Institute, University of Utah, 2000 Circle of Hope, Salt Lake City, UT 84112 USA

**Keywords:** Ponatinib, BCR-ABL, Chronic myeloid leukemia, Philadelphia chromosome, Drug safety, Electrocardiography

## Abstract

**Purpose:**

Cardiac dysfunction, particularly QT interval prolongation, has been observed with tyrosine kinase inhibitors approved to treat chronic myeloid leukemia. This study examines the effects of ponatinib on cardiac repolarization in patients with refractory hematological malignancies enrolled in a phase 1 trial.

**Methods:**

Electrocardiograms (ECGs) were collected at 3 dose levels (30, 45, and 60 mg) at 6 time points. Electrocardiographic parameters, including QTc interval, were measured, and 11 morphological analyses were conducted. Central tendency analyses of ECG parameters were performed using time-point and time-averaged approaches. All patients with at least 2 baseline ECGs and 1 on-treatment ECG were included in the analyses. Patients with paired ECGs and plasma samples were included in the pharmacokinetic/pharmacodynamic analysis to examine the relationship between ponatinib plasma concentration and change from baseline in QT intervals.

**Results:**

Thirty-nine patients at the 30-, 45-, and 60-mg dose levels were included in the central tendency and morphological analyses. There was no significant effect on cardiac repolarization, as evidenced by non-clinically significant mean QTcF changes from baseline of −10.9, −3.6, and −5.0 ms for the 30-, 45-, and 60-mg dose levels, respectively. The morphological analysis revealed 2 patients with atrial fibrillation and 2 with T wave inversion. Seventy-five patients were included in the pharmacokinetic/pharmacodynamic analysis across all dose levels. The slope of the relationship for QTcF versus plasma ponatinib concentration was not positive (−0.0171), indicating no exposure–effect relationship.

**Conclusions:**

Ponatinib is associated with a low risk of QTc prolongation in patients with refractory hematological malignancies.

## Introduction

Ponatinib (AP24534) is a novel, synthetic, orally administered, multi-targeted tyrosine kinase inhibitor (TKI) and a potent pan–BCR-ABL inhibitor [1–3]. The product of a computational and structure-based approach to the design of a small-molecule TKI, ponatinib binds with high affinity to the active site of BCR-ABL and renders binding less susceptible to any single amino acid substitution [1]. Ponatinib contains an unique carbon–carbon triple bond linkage that avoids the steric hindrance to other drugs caused by the bulky isoleucine residue at position 315 in the T315I mutant.

Based on the results in patients with chronic myeloid leukemia (CML) and Philadelphia chromosome–positive acute lymphoblastic leukemia (Ph^+^ ALL) in phase 1 testing and phase 2 clinical trials [4, 5], ponatinib (45 mg once daily) has been approved in the United States for the treatment of patients with CML and Ph^+^ ALL that is resistant or intolerant to prior TKI therapy [6].

Cardiac dysfunction has been noted with other TKIs approved for the treatment of patients with CML. For example, the imatinib prescribing information includes a warning regarding congestive heart failure and left ventricular dysfunction [7]. The nilotinib prescribing information includes “QT prolongation” as a boxed warning [8], and the dasatinib prescribing information carries “QT prolongation” as a precaution [9]. During phase 1 testing of ponatinib, treatment-related QTc prolongation was observed in 4 % of patients [4].

The QT interval is a measure of the duration of the electrical depolarization and repolarization of the ventricles of the heart and serves as a surrogate marker for the risk of torsades de pointes, which can lead to sudden death. The International Conference on Harmonisation E14 guidelines [10] outline requirements for studies of the effects of drugs on the QT interval. Specifically, the ideal QT study would include a placebo and control drug along with evaluation of a supratherapeutic dose. However, it is not often possible to implement such a study design with cancer patients, particularly the use of a placebo and positive controls.

The cardiac safety of ponatinib was initially investigated in an in vitro assay conducted in human embryonic kidney cells stably expressing the hERG potassium channel. In this study, 5 ponatinib doses were compared with the positive control cisapride (unpublished data, ARIAD Pharmaceuticals, Cambridge, MA). This preclinical study revealed that ponatinib inhibits hERG current, which is implicated in the prolongation of cardiac repolarization, at concentrations above 1 μM, which is substantially in excess of the steady-state ponatinib maximal concentrations (C_max_) observed in patients treated at the clinical dose of 45 mg orally once daily (geometric mean, 77.4 ng/mL or 0.145 μM) [4]. The cardiac safety of ponatinib was also investigated in vivo in 4 conscious telemetered dogs (unpublished data, ARIAD Pharmaceuticals, Cambridge, MA). In this study, dogs received vehicle and 3 doses of ponatinib (2, 5, and 10 mg/kg) administered 1 week apart; electrocardiographic (ECG), heart rate, and arterial pressure measurements were taken to assess the effects of ponatinib on cardiovascular parameters. This in vivo study showed that oral administration of single doses of ponatinib up to 10 mg/kg was not associated with biologically relevant effects on cardiac or circulatory function.

This report is a safety analysis of the phase 1 trial focused on the potential effects of ponatinib on cardiac repolarization in patients with refractory hematological malignancies.

## Methods

### Study design

The design of this phase 1 trial has been previously described [4]. There were 7 dose levels, with doses ranging from 2 to 60 mg. The primary objective was to determine the maximal tolerated dose, and secondary objectives included safety/tolerability, anti-leukemia activity, and pharmacokinetics (PK)/pharmacodynamics (PD). An additional secondary end point was introduced through a protocol amendment allowing analysis of ECG parameters including the QTc interval, which was primarily assessed using the Fridericia-corrected method (QTcF). The protocol amendment, under which all patients included in the central tendency and outlier analyses were evaluated, required baseline QTc to be less than 450 ms and prohibited concomitant use of medications known to prolong the QTc interval.

All patients provided signed informed consent. The protocol, amendments, and consent forms were approved by the institutional review board at each center. The study was conducted in accordance with the Guidelines for Good Clinical Practice and the Declaration of Helsinki.

### Analysis population

All patients in the 3 dose groups (30, 45, and 60 mg) with at least 2 available baseline ECGs and 1 on-treatment ECG were included in the central tendency and outlier analyses of ECG parameters (39 of 57 patients at these dose levels met these criteria). All patients across all dose levels with paired ECG and plasma concentrations for ponatinib were included in the PK/PD analysis (75 of 81 patients met these criteria).

### ECG evaluation

Resting 12-lead ECGs were collected across the 3 dose levels (30, 45, and 60 mg) and at 6 time points: baseline (day 1, in triplicate), predose; day 15, predose (single); day 29 (cycle 2/day 1), predose (in triplicate); day 29, 2 h post-dose (in triplicate); day 29, 4 h post-dose (in triplicate); and day 29, 6 h post-dose (in triplicate). Patients were supine and at rest during ECG recording, which was performed from all 12 leads simultaneously for 10 s. Electrocardiograms were recorded using GE MAC1200 ECG recorders (version 6.1) at each study site and transmitted to a central laboratory for analysis conducted by a cardiologist.

Six cardiac interval durations were measured: heart rate, PR interval, QRS interval, QT interval, QTcF, and Bazett-corrected QT (QTcB). In addition, 11 morphological analyses were conducted to identify the onset of new morphological abnormalities: atrial fibrillation and atrial flutter, second- and third-degree heart block, complete left and right bundle branch block, ST-segment change (elevation and depression separately), wave abnormalities (negative T waves only), myocardial infarction pattern, and abnormal U waves.

### Statistical analysis of ECG parameters: central tendency analysis and outlier analysis

The central tendency analysis of all ECG interval parameters, defined as a change from baseline to post-treatment time points (except for cycle 1/day 15, predose), was performed using 2 approaches: time point and time averaged. For the time-point analysis, 3 ECGs were to be collected at each time point (baseline and 4 post-treatment visits); however, post-dose time points with only 2 ECGs were included in this analysis. The data from the 2 or 3 ECGs were averaged to provide a single set of ECG intervals for each time point. Data were summarized using descriptive statistics. Changes from baseline to 4 post-treatment time points were described with data-based (not model-based) 2-sided 90 % CI statistics. For QTc measurements, the QTcF method was the primary measurement; QTcB was considered secondary, provided for historical purposes only. For the time-averaged analysis, baseline time points were averaged and the value obtained was subtracted from the mean of all combined 4 post-treatment ECG time points.

Outlier or categorical analysis was also performed to identify patients who experienced a significant effect on any ECG interval parameter (heart rate, PR interval, QRS interval, QT interval, QTcF, and QTcB) that would not be revealed by the central tendency analysis and should be considered exploratory in nature. This analysis used a time-averaged approach that compared the baseline ECG interval value with all post-treatment ECG time points, and then, the value that represented the greatest positive change from baseline was chosen to determine whether each patient fell into the outlier criterion. For heart rate, both the largest negative and positive value compared with baseline was chosen.

### Pharmacokinetic/pharmacodynamic evaluations

Plasma samples were collected concomitantly with ECG assessments. A linear mixed-effects modeling approach was used to quantify the relationship between the plasma concentration of ponatinib and the change from baseline in QT intervals. This model was used to estimate the population slope and the standard error of the slope of the relationship between the change from baseline in QTc intervals and plasma concentrations of ponatinib. As this model is meant solely to determine the relationship of QTc change with the degree of change in exposure, the time points are not relevant; therefore, all plasma concentration and time point QTc pairs were used irrespective of the time point and the dose group from which such pairs were taken. A linear relationship was declared if the *P* value of the slope was less than 0.05.

## Results

### Patient characteristics

Thirty-nine patients who received 30–60 mg of ponatinib once daily were included in the primary cardiac safety analysis. The demographic characteristics are summarized in Table 1. In the phase 1 clinical study, geometric mean (range) values of C_max_ for ponatinib measured at steady state (day 29) were 64.6 (35.9–94.8) ng/mL at 30 mg, 77.4 (34.3–179) ng/mL at 45 mg, and 97.5 (54.3–231) ng/mL at 60 mg [4]. As of March 23, 2012, 7 patients had experienced 1 or more treatment-related adverse events in the cardiac disorders MedDRA system organ class (2 left ventricular dysfunction, 2 tricuspid valve incompetence, and 1 each aortic valve sclerosis, atrial fibrillation, cardiac failure congestive, cardiomegaly, cardiomyopathy, left ventricular hypertrophy, palpitations, and pericardial effusion). Most of these events were grade 1 or 2 in severity. Three patients experienced treatment-related adverse events of QT prolongation (grade 2 or 3).

**Table 1:** Demographic characteristics of patients included in the cardiac analysis

Characteristics	Ponatinib 30–60 mg (*N* = 39)
Age	
Median (range), years	49.0 (27–85)
≥65 years, *n* (%)	8 (20.5)
Gender, *n* (%)	
Male	26 (66.7)
Female	13 (33.3)
ECOG performance status, *n* (%)	
0	20 (51.3)
1	15 (38.5)
2	4 (10.3)
Median time (range) from diagnosis to treatment, years	4.8 (0.6, 23.5)
Diagnosis, *n* (%)	
Chronic-phase CML	25 (64.1)
Accelerated-phase CML	1 (2.6)
Blast-phase CML	4 (10.3)
Ph^+^ ALL	4 (10.3)
AML	5 (12.8)
Prior number of TKIs, *n* (%)	
0	0 (0.0)
1	3 (7.7)
2	12 (30.8)
≥3	19 (48.7)
Missing	5 (12.8)

*AML* acute myeloid leukemia, *CML* chronic myeloid leukemia, *ECOG* Eastern Cooperative Oncology Group, *Ph* ^*+*^ *ALL* Philadelphia chromosome–positive acute lymphoblastic leukemia, *TKIs* tyrosine kinase inhibitors

### Time-averaged central tendency analysis

The mean changes from baseline in heart rate across ponatinib dose levels (+3.5 bpm, −3.3 bpm, and +1.0 bpm for 30-, 45-, and 60-mg dose levels, respectively) were not clinically significant; there were 2 tachycardia outliers (1 at the 45-mg dose level and 1 at the 60-mg dose level) and no bradycardia outliers (Table 2). The effects on atrioventricular conduction, as measured by mean change from baseline in the PR interval (−0.4, −3.6, and −0.7 ms for the 30-, 45-, and 60-mg dose levels, respectively), were not clinically significant, and there were no outliers (Table 2). The time-averaged mean change from baseline across the ponatinib 30-, 45-, and 60-mg dose levels for QRS interval duration showed a change of −0.8, +1.3, and +3.6 ms (Table 2). These changes are unlikely to be clinically relevant, and there were no outliers. A small effect on QRS interval cannot be ruled out at 60 mg due to the small sample size (12 patients); however, the recommended dose of ponatinib is 45 mg. The time-point analysis showed no signal of any effect. There was no significant effect on cardiac repolarization, as demonstrated by the non-clinically significant change in QTcF across the doses examined. The mean QTcF changes from baseline were −10.9, −3.6, and −5.0 ms for the 30-, 45-, and 60-mg dose levels, respectively.

**Table 2:** Electrocardiographic interval parameters (time-averaged central tendency analysis), outlier analysis, and morphological abnormalities, by dose level

	Ponatinib 30 mg (*n* = 6)	Ponatinib 45 mg (*n* = 21)	Ponatinib 60 mg (*n* = 12)
*Electrocardiographic interval parameters and outlier analysis*			
Heart rate, mean change from baseline, bpm	3.5	−3.3	1.0
Heart rate tachycardic outliers, *n* (%)	0 (0)	1 (5)	1 (8)
PR interval, mean change from baseline, ms	−0.4	−3.6	−0.7
PR interval outliers, *n* (%)	0 (0)	0 (0)	0 (0)
QRS interval, mean change from baseline, ms	−0.8	1.3	3.6
QRS interval outliers, *n* (%)	0 (0)	0 (0)	0 (0)
QT interval, mean change from baseline, ms	−13.4	3.3	−4.6
QTcF, mean change from baseline, ms	−10.9	−3.6	−5.0
QTcF >500 ms, *n* (%)	0 (0)	1 (5)	0 (0)
QTcF >60 ms change from baseline, *n* (%)	0 (0)	0 (0)	1 (8)
QTcF 30- to 60-ms change from baseline, *n* (%)	0 (0)	3 (14)	0 (0)
QTcB^a^, mean change from baseline, ms	−9.2	−7.4	−4.9
*Morphological abnormalities, n (%)*			
Atrial fibrillation	1 (17)	0 (0)	1 (8)
T wave inversion	0 (0)	1 (5)	1 (8)
Atrial flutter	0 (0)	0 (0)	0 (0)
Myocardial infarction	0 (0)	0 (0)	0 (0)
Second- or third-degree heart block	0 (0)	0 (0)	0 (0)
RBBB or LBBB	0 (0)	0 (0)	0 (0)
ST-segment elevation or depression	0 (0)	0 (0)	0 (0)
Abnormal U waves	0 (0)	0 (0)	0 (0)

*bpm* beats per minute, *ms* milliseconds, *QTcF* Fridericia-corrected QT, *QTcB* Bazett-corrected QT, *RBBB* right bundle branch block, *LBBB* left bundle branch block

^a^The Bazett correction method is often less reliable than the Fridericia correction method; QTcB is provided for historical purposes only

The findings from the outlier analysis of absolute QTcF duration identified few patients experiencing QTcF prolongation (Table 2). One patient at the 45-mg dose level (5 %) had a QTcF >500 ms; 1 patient at the 60-mg dose level (8 %) had a change in QTcF >60 ms from baseline; and 3 patients at the 45-mg dose level (14 %) had a 30- to 60-ms change in QTcF from baseline (Table 2). The patient at the 45-mg dose level with a QTcF >500 ms had a C_max_ of 57.6 ng/mL, which was below the geometric mean C_max_ at the 45-mg dose level (77.4 ng/mL) [4]. This patient was receiving concomitant Darvocet (acetaminophen and propoxyphene), a medication known to prolong the QTc interval.

### Morphological analysis

Atrial fibrillation and T wave inversion were observed in 2 chronic-phase CML patients each (Table 2). Three of these 4 patients had a history of cardiovascular disease (e.g., stroke, hypertension, intermittent sinus bradycardia, and palpitations), suggesting that these morphological abnormalities may reflect the patient population being studied rather than representing an effect of the study medication. Given their age (median, 49 years) and ECOG performance status, the patients in this study are representative of the patient population that will be treated with ponatinib in the clinic. The fourth patient was taking concomitant moxifloxacin, a medication known to be associated with cardiac arrhythmias (in violation of the study protocol) [11].

### Time-point central tendency analysis

**Fig. 1:**
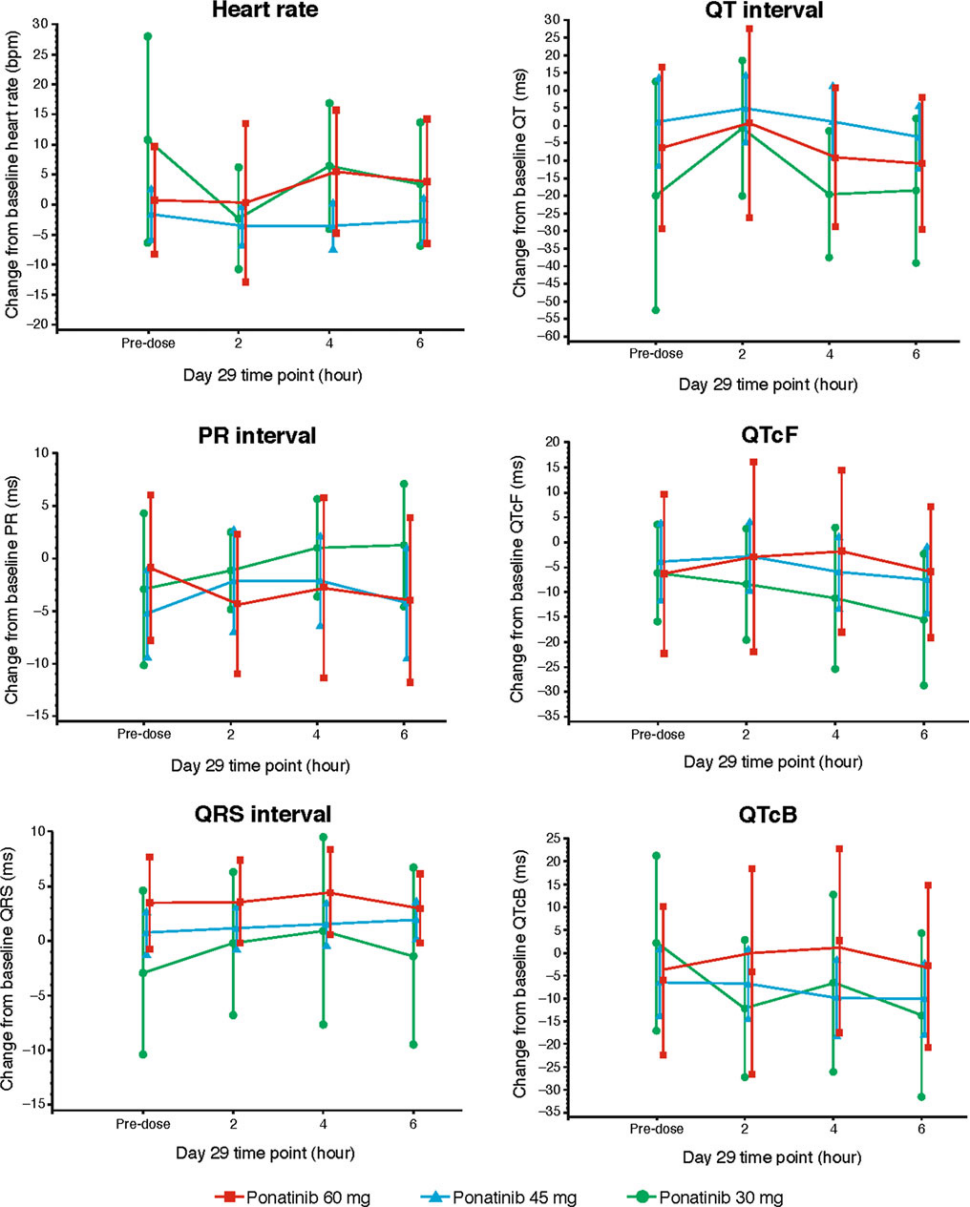
Mean (±90 % CI) change from baseline in electrocardiographic interval parameters for 4 on-treatment time points (time-point central tendency analysis). *CI* confidence interval, *bpm* beats per minute, *ms* milliseconds, *QTcF* Fridericia-corrected QT, *QTcB* Bazett-corrected QT

Time-point analysis of 6 ECG parameters revealed no effect of ponatinib across 4 on-treatment time points (Fig. 1). These time points were selected based on the steady-state C_max_ of ponatinib; therefore, these findings indicate that even at the highest concentrations, ponatinib had no significant effect on the ECG parameters analyzed (Fig. 1).

### Pharmacokinetic/pharmacodynamic analysis

**Fig. 2:**
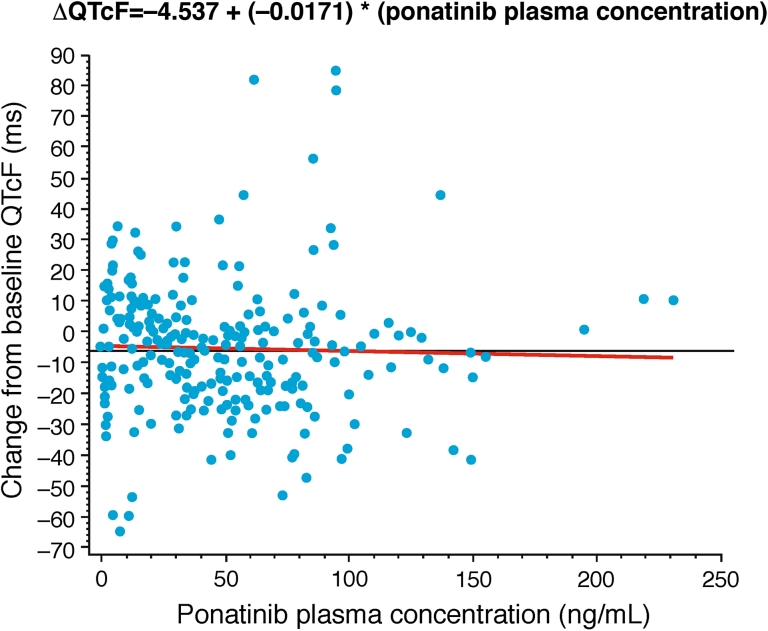
QTcF change from baseline by ponatinib plasma concentration across 7 dose levels (*N* = 69). *QTcF* Fridericia-corrected QT, *ms* milliseconds

Sixty-nine of the total 81 patients enrolled in this phase 1 study had paired baseline/post-baseline time-matched PK-ECG data and were included in the concentration delta QTc mixed model analysis. The slope of the relationship for QTcF versus plasma ponatinib concentration was not positive (Table 3; Fig. 2), indicating no exposure–effect relationship. The estimated QTcF mean change at C_max_ was −6.4 ms at the 60-mg dose level and −6.2 at the 45-mg dose level (Table 4).

**Table 3:** Change from baseline versus ponatinib plasma concentration

	QTc parameter	
	QTcF	QTcB
Slope of plasma concentration effect on ΔQTc	−0.0171	−0.0091
Standard error of plasma concentration effect on ΔQTc	0.0321	0.0358
*P* value slope of plasma concentration effect on ΔQTc	0.5958	0.7997
Overall model fit	<0.0001	<0.0001

*QTcF* Fridericia-corrected QT, *QTcB* Bazett-corrected QT

**Table 4:** Estimates from linear mixed model QTcF and QTcB

	Ponatinib 30 mg (*n* = 6)		Ponatinib 45 mg (*n* = 21)		Ponatinib 60 mg (*n* = 12)		Ponatinib 30–60 mg (*N* = 39)	
QTc parameter	QTcF	QTcB	QTcF	QTcB	QTcF	QTcB	QTcF	QTcB
Predicted ΔQTc at average C_max_, ms	−5.5888	−5.6112	−6.2318	−5.9544	−6.4421	−6.0666	−6.1885	−5.9313
One-sided upper 95 % confidence bound of predicted ΔQTc, ms	−1.3242	−0.7051	−1.1137	−0.0432	−0.9124	0.3126	−1.1486	−0.1096

*QTcF* Fridericia-corrected QT, *QTcB* Bazett-corrected QT, *ms* milliseconds

## Discussion

This analysis of QTc intervals in patients with refractory hematological malignancies who received daily doses of 30, 45, or 60 mg of ponatinib in a phase 1 clinical trial revealed no significant effect of ponatinib on cardiac repolarization. The recommended dose of ponatinib is 45 mg. Initial characterization of cardiac safety, including QT prolongation, was previously described across all 81 patients included in this phase 1 trial [4]. Although dose-limiting toxicities identified in phase 1 did not include cardiovascular findings, among the adverse events reported in the trial (*n* = 81), 3 patients (4 %) experienced treatment-related QT prolongation: 1 patient each at the 2-, 4-, and 45-mg dose levels. Of these 3 patients, 2 (3 %) experienced grade 3 treatment-related QT prolongation (at the 4- and 45-mg dose levels). All 3 patients had low steady-state C_max_ (4.5–57.6 ng/mL), suggesting that QT prolongation was not due to increased ponatinib exposure. Two of the 3 patients were enrolled before protocol amendment, and all 3 patients were found to have prolongation of QTc at baseline or to have received concomitant medications known to be associated with QTc prolongation. There were no clinical consequences of the ECG findings in these patients.

The results of this cardiac analysis suggest that ponatinib is associated with a low risk of QTc prolongation. Other targeted agents approved for the treatment of CML have been found to be associated with cardiac toxicities [7–9]. Imatinib has been associated with left ventricular dysfunction and heart failure, particularly in patients with comorbidities and risk factors [7, 12]. In the phase 3 International Randomized Study of Interferon and STI571 (IRIS) in 1,106 patients with newly diagnosed Ph^+^ CML, severe cardiac failure and left ventricular dysfunction were observed in 0.7 % of patients taking imatinib compared with 0.9 % of patients taking interferon alfa plus cytarabine [7, 13, 14]. The dasatinib prescribing information carries QT prolongation as a precaution. In a phase 1 trial (NCT01392703) in 75 healthy subjects, a clear QT prolongation effect was not detected [15]. However, this adverse event emerged in a phase 3 trial conducted in patients newly diagnosed with CML: QTc intervals between 450 and 500 ms were observed in 2 % of the patients taking dasatinib, compared with 4 % of patients taking imatinib [16]. The nilotinib prescribing information includes a boxed warning regarding QT prolongation [8]. Results of ECG analyses conducted on about 400 patients with CML who participated in a phase 1/2 trial (NCT00109707) showed a significant association between nilotinib concentration and a change from baseline in QTcF, indicating a prolongation of the QTc interval associated with nilotinib [17–19]. A modest linear correlation between nilotinib concentration and a change from baseline in QTcF along with a higher incidence of developing ischemic heart disease in the nilotinib arms was also found in the phase 3 trial Evaluating Nilotinib Efficacy and Safety in Clinical Trials—Newly Diagnosed Patients (ENESTnd) conducted in patients with newly diagnosed CML [20, 21]. Finally, the effects of bosutinib on cardiac repolarization were studied in a randomized, crossover, placebo- and moxifloxacin-controlled study. In the healthy adult subjects enrolled in this study, therapeutic and supratherapeutic bosutinib exposures were not associated with QTc prolongation [22].

This study had 2 primary limitations. First, the study was not designed as a true thorough QT study as outlined by the International Conference on Harmonisation of Technical Requirements for Registration of Pharmaceuticals for Human Use E14 guidelines. However, it is worthwhile noting that formal thorough QT studies are difficult, if not impossible, to conduct ethically in this patient population owing to the requirement for placebo and positive controls. Second, the number of subjects enrolled in the study was relatively small, particularly for dose-level analyses. Unlike the healthy subject QTc analysis conducted with nilotinib, this study evaluated the cardiac effects of ponatinib in a patient population with refractory hematological malignancies, which may increase confidence that these results are consistent with what will be seen in clinical practice.

The results of this QTc analysis in CML patients treated with ponatinib at clinically relevant doses suggest that ponatinib is associated with a low risk of QTc prolongation.
